# Congestive Heart Failure versus Inflammatory Carcinoma in Breast

**DOI:** 10.1155/2014/815896

**Published:** 2014-04-07

**Authors:** A. Alikhassi, R. Omranipour, Z. Alikhassy

**Affiliations:** ^1^Breast Clinic, Cancer Institute, Tehran University of Medical Science, Tehran 1419733141, Iran; ^2^Emergency Department, Isfahan University, Iran

## Abstract

Inflammatory breast cancer is a rare highly malignant form of breast cancer. Clinical signs and symptoms with histologic examination usually confirm the diagnosis. There are rare reports of breast edema of congestive heart failure which were difficult to differentiate from inflammatory carcinoma. The differential becomes more difficult when congestive heart failure is associated with unilateral breast edema. We present a case of a 70-year-old woman with congestive heart failure associated with unilateral breast edema and skin thickening simulating inflammatory breast carcinoma on mammography.

## 1. Introduction

Congestive heart failure is a common disease especially in elderly population. Its common presentations are well known such as dyspnea, fatigue, weakness, swelling in legs, increased need to urinate at night, and lack of appetite, but familiarizing with the rare presentations of this common disease is also important to prevent unnecessary treatments, cost, and morbidity. We report a rare case of unilateral breast enlargement in an elderly woman with congestive heart failure who was clinically suspicious of inflammatory breast cancer.

## 2. Case Presentation

A 70-year-old retired woman presented in breast clinic of cancer institute with complaint of gradual enlargement of right breast with peau d'orange appearance and pitting edema without accompanying arm edema and exertional dyspnea, which happened gradually during the last few months. She did not have fever or dermal erythema. She did not give any history of breast trauma or any previous surgery. She had a past medical history of congestive heart failure (EF 30%).

On clinical exam, the patient was cooperative and tachypnoeic and had heart rate of 70/min, regular rhythm, and blood pressure of 130/70 mmHg. There were bilateral crepitations of the lungs, more in the right side. There were bilateral legs pitting edema. Abdominal exam was normal.

Clinical breast examination showed an enlarged right breast mainly in dependent part with diffusely thickened peau d'orange skin. There was no nipple discharge, nipple retraction, palpable breast mass, or palpable axillary lymphadenopathy.

**Figure 1:**
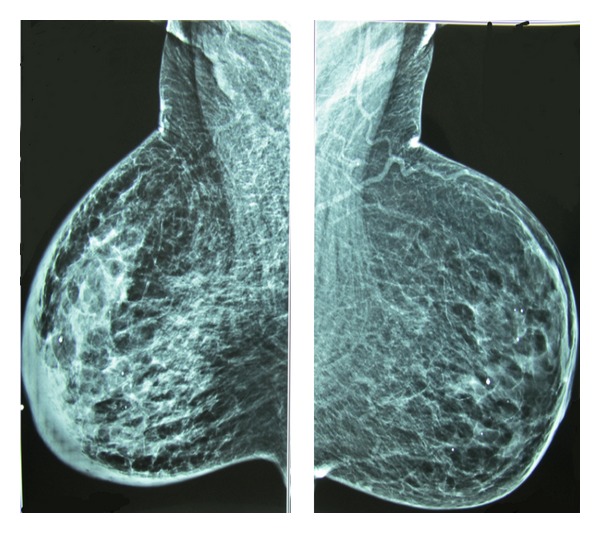
Bilateral mammography (MLO view).

**Figure 2:**
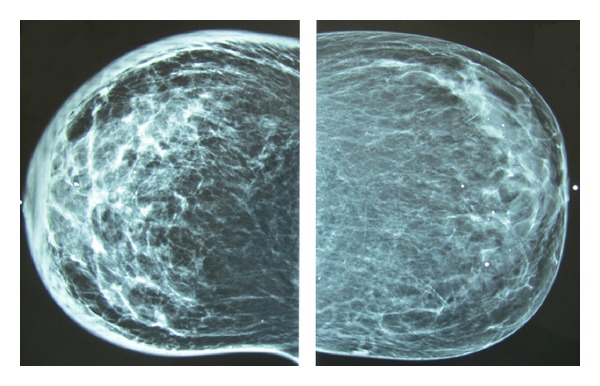
Bilateral mammography (CC view).

Full digital mammography revealed skin thickening, cooper ligaments thickening, and edema without any apparent mass. Some punctate benign-type microcalcifications also spread in both breasts (Figures 1 and 2).

**Figure 3:**
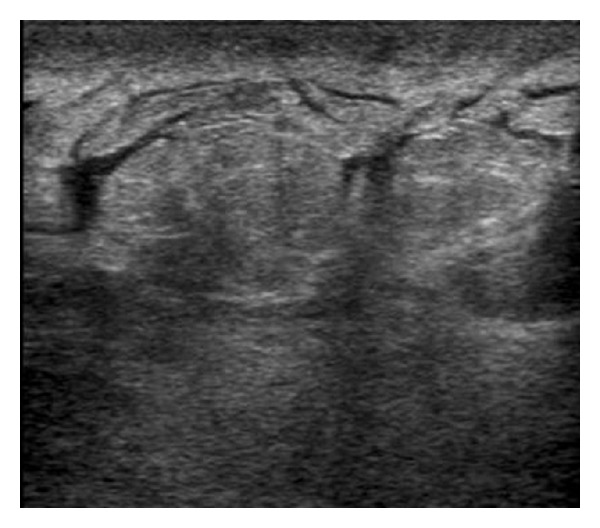
Breast ultrasound.

Ultrasound showed skin thickening, trabecular prominancy, and generalized mild tissue distortion due to edema without apparent mass (Figure 3).

**Figure 4:**
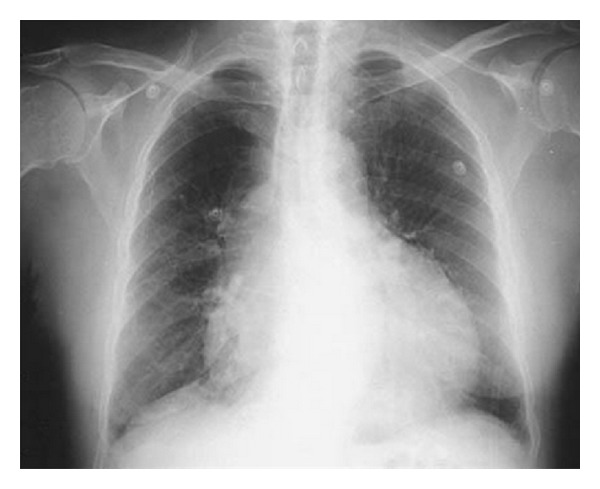
CXR.

Her chest X-ray showed cardiac enlargement and right-side pleural effusion (Figure 4).

**Figure 5:**
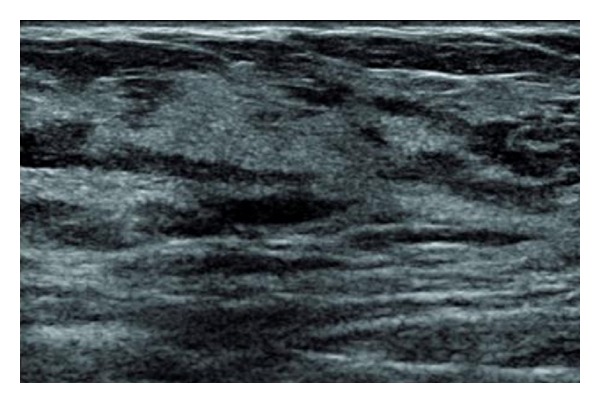
Follow-up ultrasound.

She was a known case of congestive heart failure with low ejection fraction. Heart failure was considered as a differential diagnosis for more important inflammatory breast cancer despite, breast edema due to heart failure is a rare entity. We recommended confirming diagnosis by tissue biopsy (skin punch biopsy for ruling out inflammatory breast cancer), but she refused. Alternatively, diuretics were prescribed and she has been on surveillance in our clinic for one year; treatment with fluid restriction and frusemide produced satisfactory diuresis and decreased symptoms. Breast size, peau d'orange skin appearance, and breast edema all decreased. Her follow-up ultrasounds also did not show any mass and proved decrease of edema and skin thickening (Figure 5). In contradiction to inflammatory breast cancer behavior, she has been fairly well during one-year followup in our clinic.

## 3. Discussion

Diagnosis of breast inflammatory carcinoma should be based on the clinical manifestations, consisting of increased warmth, erythema, and the classic peau d'orange (skin of an orange) appearance of the thickened skin. The latter is named because of the accentuation of the depressions around the hair follicles caused by skin edema. However, this appearance can be mimicked by benign inflammatory processes. It is difficult to make the diagnosis and frequently biopsy is required. Antibiotic therapy may improve the clinical status in both benign and malignant diseases [1].

Histologically, inflammatory carcinoma represents diffuse early invasion of the dermal lymphatics by an aggressive form of infiltrating carcinoma. In its pure form, the underlying primary lesion is not frequently evident. Fortunately it is fairly uncommon and comprises approximately 0.5% of invasive breast cancers [1]. Although the incidence of inflammatory cancer increases with age, its relative frequency is the highest in young women, peaking around 30 years [1]. In spite of some improvements in treatment (i.e., chemotherapy, mastectomy, and radiation therapy), patients with inflammatory carcinoma generally have a poor prognosis. Pure inflammatory carcinoma appears to carry a worse prognosis compared with infiltrating ductal carcinoma with secondary local skin invasion [2]. Inflammatory carcinoma appears to have slightly more tendency to left breast [3].

### 3.1. Mammographic Appearance

The mammographic appearance of inflammatory carcinoma is indistinguishable from other processes that cause skin thickening and edema. It is unusual to find mass or tumor-associated calcifications [2].

Inflammatory carcinoma in association with an underlying mass is probably a separate entity representing a later-stage progression of an infiltrating tumor mass with secondary invasion of the dermal lymphatics. As any other process that overwhelms the lymphatic drainage, diffuse trabecular thickening and overall increase in X-ray attenuation may produce diffuse increase in radiographic density of the affected breast [1, 2].

### 3.2. Ultrasound Appearance

Ultrasound has no major role in evaluation of inflammatory carcinoma unless an abscess or a deep mass is suspected. Inflammatory carcinoma produces nonspecific skin thickening, which cannot be distinguished from other processes by ultrasound. Ultrasound cannot differentiate plain and inflammatory edema from actual tumor infiltration unless an underlying abscess is evident and is confirmed by aspiration. The diagnosis of inflammatory cancer is based on the clinical constellation and histopathologic confirmation [3, 4].

There are rare reports of difficulties in differentiating this particular type of breast malignancy from congestive heart failure (CHF). This difficulty arises when CHF is associated with unilateral breast edema and skin thickening [5]. However, inflammatory breast carcinoma has distinctive histologic and microscopic characteristics allowing the establishment of a proper diagnosis [4]. Congestive cardiac failure is a common condition and its frequent manifestation is well known. However, breast enlargement as a manifestation of cardiac failure is quite uncommon, and the condition is very rarely documented in the literature as it is usually bilateral, since systemic diseases usually give rise to bilateral abnormalities [6, 7].

In review of the previous literature, we found five reports of heart failure simulating IBC (Table 1).

Congestive heart failure and any process that obstructs cardiac venous return, such as mediastinal tumor (superior vena cava obstruction), can cause breast edema. Dermatological causes such as Psoriasis may have the same manifestation; this etiology should be suspected, especially when the thickening is bilateral. Milroy's disease, the congenital absence of the lymphatics, can also produce skin thickening [5, 8].

A list of differential diagnoses of unilateral diffuse breast edema is shown as follows.

Diffuse breast edema and thickening differential diagnosis:

 inflammatory breast carcinoma,
 congestive heart failure,
 mastitis,
 leukemia,
 lymphoma,
 secondary to radiotherapy,
 nephrotic syndrome,
 lymphoma,
 progressive systemic sclerosis,
 superior vena cava syndrome,
 pemphigus and other skin conditions.

If changes are unilateral, the possibility of carcinoma is of particular concern, since systemic diseases usually give rise to bilateral abnormalities. Breast cancer is the primary diagnosis of unilateral breast enlargement; as a result the patient undergoes all the expensive investigative procedures, although it may be due to a benign process like tendency to lie on one side and causing dependent edema [7, 9]. Pitting edema of the breast in the absence of palpable mass is helpful to differentiate the diagnosis. Obviously, by treatment of heart failure the breast edema should decrease [6, 10]. It should be mentioned that atrophic breast of elderly women is more susceptible to edema rather than young women in the reproductive age [3].

## 4. Conclusion

It is important to recognize this rare presentation of a very common condition just to avoid unnecessary investigations. Breast edema should be considered as a rare presentation of congestive heart failure.

## Figures and Tables

**Table 1:** Previous reports of unilateral breast edema in cardiac heart failure mimicking inflammatory breast cancer.

Authors	Publication year	Number of patients
Lindhardt [11]	1981	1
M$\ddot{\text{u}}$ller and Koehler [12]	1984	2
Doyle and Fracr [13]	1991	1
Pluchinotta et al. [5]	1994	2
Oraedu et al. [14]	2001	1
